# PRE-OPerative ECHOcardiograhy for prevention of cardiovascular events after non-cardiac surgery in intermediate- and high-risk patients: protocol for a low-interventional, mixed-cohort prospective study design (PREOP-ECHO)

**DOI:** 10.1186/s13063-022-06701-2

**Published:** 2022-09-14

**Authors:** Eun Kyoung Kim, Hong-Mi Choi, Eui-Young Choi, Hye Sun Lee, Goeun Park, Dong Woo Han, Sang-Eun Lee, Chan Seok Park, Ji-won Hwang, Jae Hyuk Choi, Mi-Na Kim, Hyung-Kwan Kim, Dae-Hee Kim, Sung-Hee Shin, Il Suk Sohn, Mi-Seung Shin, Jin Oh Na, Iksung Cho, Sun Hwa Lee, Yong Hyun Park, Tae-Ho Park, Kye Hun Kim, Goo-Young Cho, Hae Ok Jung, Dae-Gyun Park, Ji Yeon Hong, Duk-Hyun Kang

**Affiliations:** 1 Division of Cardiology, Department of Medicine, Cardiovascular Imaging Center, Heart Vascular Stroke Institute, Samsung Medical Center, Sungkyunkwan University School of Medicine, Seoul, South Korea; 2 Department of Cardiology, Cardiovascular Center, Seoul National University Bundang Hospital, Seoul National University College of Medicine, Seongnam, Gyeonggi-do South Korea; 3 Division of Cardiology, Gangnam Severance Hospital, Yonsei University College of Medicine, 211 Eonju-Ro, Gangnam-Gu, Seoul, 06273 South Korea; 4 Biostatistics Collaboration Unit, Yonsei University College of Medicine, Seoul, South Korea; 5 Department of Anesthesiology and Pain Medicine, Yonsei University College of Medicine, Seoul, South Korea; 6 Division of Cardiology, Department of Internal Medicine, Ewha Womans University College of Medicine, Seoul, South Korea; 7 Department of Cardiology, Bucheon St. Mary’s Hospital, The Catholic University College of Medicine, Bucheon, South Korea; 8 Division of Cardiology, Department of Medicine, Ilsan Paik Hospital, Inje University School of Medicine, Goyang, South Korea; 9 Division of Cardiology, Hallym University Dongtan Sacred Heart Hospital, Hwaseong, South Korea; 10 Division of Cardiology, Korea University Anam Hospital, Seoul, South Korea; 11 Division of Cardiology, Cardiovascular Center, Department of Internal Medicine, Seoul National University College of Medicine, Seoul, South Korea; 12 Department of Cardiology, Asan Medical Center, Ulsan University College of Medicine, Seoul, South Korea; 13 Division of Cardiology, Inha University Medical Center, Incheon, South Korea; 14 Department of Cardiology, Cardiovascular Center, Kyung Hee University Hospital at Gangdong, Seoul, South Korea; 15 Division of Cardiology, Department of Internal Medicine, Gil Medical Center, Gachon University College of Medicine, Incheon, South Korea; 16 Cardiovascular Center, Korea University Guro Hospital, Seoul, South Korea; 17 Division of Cardiology, Severance Cardiovascular Hospital, Yonsei University College of Medicine, Seoul, South Korea; 18 Division of Cardiology, Department of Internal Medicine, Jeonbuk National University Medical School, Jeonju, South Korea; 19 Division of Cardiology, Department of Internal Medicine, Pusan National University Yangsan Hospital, Yangsan, South Korea; 20 Department of Cardiology, Dong-A University College of Medicine, Busan, South Korea; 21 Department of Cardiovascular Medicine, Chonnam National University Hospital, Gwangju, South Korea; 22 Division of Cardiology, Department of Internal Medicine, Seoul St. Mary’s Hospital, College of Medicine, The Catholic University of Korea, Seoul, South Korea; 23 Department of Internal Medicine, Kangdong Sacred Heart Hospital, Hallym University, Seoul, South Korea; 24 Division of Cardiology, Hallym University Sacred Heart Hospital, Hallym University College of Medicine, Anyang, South Korea

**Keywords:** Echocardiography, Noncardiac surgery, Trial design

## Abstract

**Background:**

Cardiac evaluation using transthoracic echocardiography before noncardiac surgery is common in real-world practice. However, evidence supporting preoperative echocardiography is lacking. This study aims to evaluate the additional benefit of preoperative echocardiography in predicting postoperative cardiovascular events (CVE) in noncardiac surgery.

**Methods:**

This study is designed as a multicenter, prospective study to assess the utility of preoperative echocardiography in patients undergoing intermediate- or high-risk noncardiac surgery. This trial comprises two studies: (1) a randomized controlled trial (RCT) for patients undergoing intermediate-risk surgery with fewer than three clinical risk factors from the revised cardiac risk index (intermediate-risk group) and (2) a prospective cohort study for patients undergoing intermediate-risk surgery with three or more clinical risk factors, or who undergo high-risk surgery regardless of the number of clinical risk factors (high-risk group). We hypothesize that the use of preoperative echocardiography will reduce postoperative CVEs in patients undergoing intermediate- to high-risk surgery through discovery of and further intervention for unexpected cardiac abnormalities before elective surgery. A total of 2330 and 2184 patients will be enrolled in the two studies. The primary endpoint is a composite of all-cause death; aborted sudden cardiac arrest; type I acute myocardial infarction; clinically diagnosed unstable angina; stress-induced cardiomyopathy; lethal arrhythmia, such as sustained ventricular tachycardia or ventricular fibrillation; and/or newly diagnosed or acutely decompensated heart failure within 30 days after surgery.

**Discussion:**

This study will be the first large-scale prospective study examining the benefit of preoperative echocardiography in predicting postoperative CVE. The PREOP-ECHO trial will help doctors identify patients at risk of postoperative CVE using echocardiography and thereby reduce postoperative CVEs.

**Trial registration:**

The Clinical Research Information Service KCT0006279 for RCT and KCT0006280 for prospective cohort study. Registered on June 21, 2021.

**Supplementary Information:**

The online version contains supplementary material available at 10.1186/s13063-022-06701-2.

## Background

Development of unexpected postoperative adverse events after noncardiac surgery, especially cardiac events, is a major concern to all clinicians including surgeons and anesthesiologists, as well as the patients and their family members. Postoperative cardiovascular events (CVE) are likely to be associated with both patients’ comorbidities and the risk of the operation and anesthesia. Up to 8% of patients experienced cardiovascular death or non-fatal myocardial infarction within 30 days after surgery in a recent prospective study [1]. Although the incidence of all-cause mortality, specifically fatal CVE, after noncardiac surgery has decreased with advanced intraoperative monitoring systems, surgical techniques, and elaborate postoperative management, the frequency of nonfatal CVEs is increasing as surgeries performed in older patients with elevated comorbidity status increase in number [2–6]. Various predictive models have been proposed to assess CVE risk following noncardiac surgery [7–11]. However, previous models only focus on hard outcomes such as fatal and non-fatal myocardial infarction (MI) and cardiac arrest, not on nonfatal but important outcomes, such as unstable angina, stress-induced cardiomyopathy, and acute decompensated heart failure. Therefore, preoperative cardiac risk assessment tools using existing guidelines have limited predictive power for common postoperative CVEs in real-world clinical practice.

Transthoracic echocardiography (TTE) has been widely used to assess the potential risk of postoperative CVE and to detect severe structural heart diseases, such as valvular heart diseases or left ventricular (LV) dysfunction, before noncardiac surgery. Even though TTE is a noninvasive, reproducible, and informative tool for evaluating individual cardiovascular conditions, most guidelines do not recommend preoperative TTE in asymptomatic patients [7, 8, 12, 13]. The benefits of echocardiographic parameters for the prediction of postoperative CVE have not yet been demonstrated in patients without symptoms or signs of cardiovascular disease, and indiscriminate use of preoperative TTE can lead to an unnecessary increase in medical costs. Interestingly, an increasing number of clinicians are using preoperative TTE, not being supported by the current guidelines [7, 8, 12, 13]. Cardiac biomarkers, such as natriuretic peptides and troponins reflecting increased LV wall stress or myocardial injury sensitively, might also have an additive role in the detection of subclinical cardiac diseases and the prognostication for the postoperative CVE [14–18]. However, current guidelines do not recommend the routine use of preoperative biomarkers to predict postoperative CVEs [7, 8, 12, 13].

To bridge this evidence gap, we designed a study entitled “PRE-OPerative ECHOcardiography for the prevention of cardiovascular events after noncardiac surgery in intermediate- and high-risk patients (PREOP-ECHO)”. We hypothesize that preoperative TTE would reduce postoperative CVEs in patients undergoing intermediate- to high-risk surgery by screening and timely addressing hidden cardiac conditions before elective noncardiac surgery. The main purpose of this study is to evaluate the additional benefit of preoperative echocardiography in predicting postoperative CVEs in noncardiac surgery.

## Trial design

### Study objectives

The purpose of this study is to compile data on preoperative TTE from the perspective of reducing postoperative CVEs within 30 days after intermediate- and high-risk noncardiac surgery. Previous risk scoring systems have focused primarily on MI and cardiac arrest as adverse cardiac events [7–11]. Therefore, this trial will encompass all clinically relevant CVEs as primary and secondary endpoints. Further, this trial will develop possible echocardiographic parameters that could be used to determine a preoperative assessment system that would assist in predicting postoperative CVEs. The results from this study will be used to develop Korean guideline for the use of preoperative echocardiography in patients who will undergo noncardiac surgery.

### Study design

Current guidelines do not recommend preoperative TTE in asymptomatic patients undergoing intermediate- or low-risk noncardiac surgery (class of recommendation [COR] III, level of evidence [LOE] C); however, preoperative TTE may be considered in patients who undergo high-risk surgery (COR IIb, LOE C) [8]. Therefore, we will divide participants enrolled in this trial into two study cohorts based on the potential risk of postoperative CVE.

**Fig. 1:**
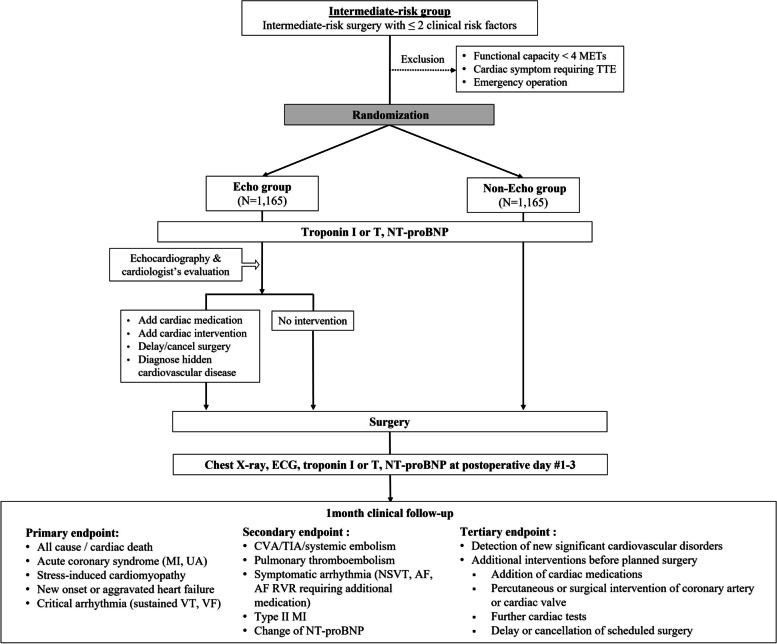
Study flow of the PREOP-ECHO trial: randomized controlled trial for the intermediate-risk group. Abbreviations. AF, atrial fibrillation; CVA, cerebrovascular accident; ECG, electrocardiography; MET, metabolic equivalent of task; MI, myocardial infarction; NSVT, nonsustained VT; NT-proBNP, N-terminal B-type natriuretic peptide; RVR, rapid ventricular response; TIA, transient ischemic attack; TTE, transthoracic echocardiography; VF, ventricular fibrillation; VT, ventricular tachycardia

**Fig. 2:**
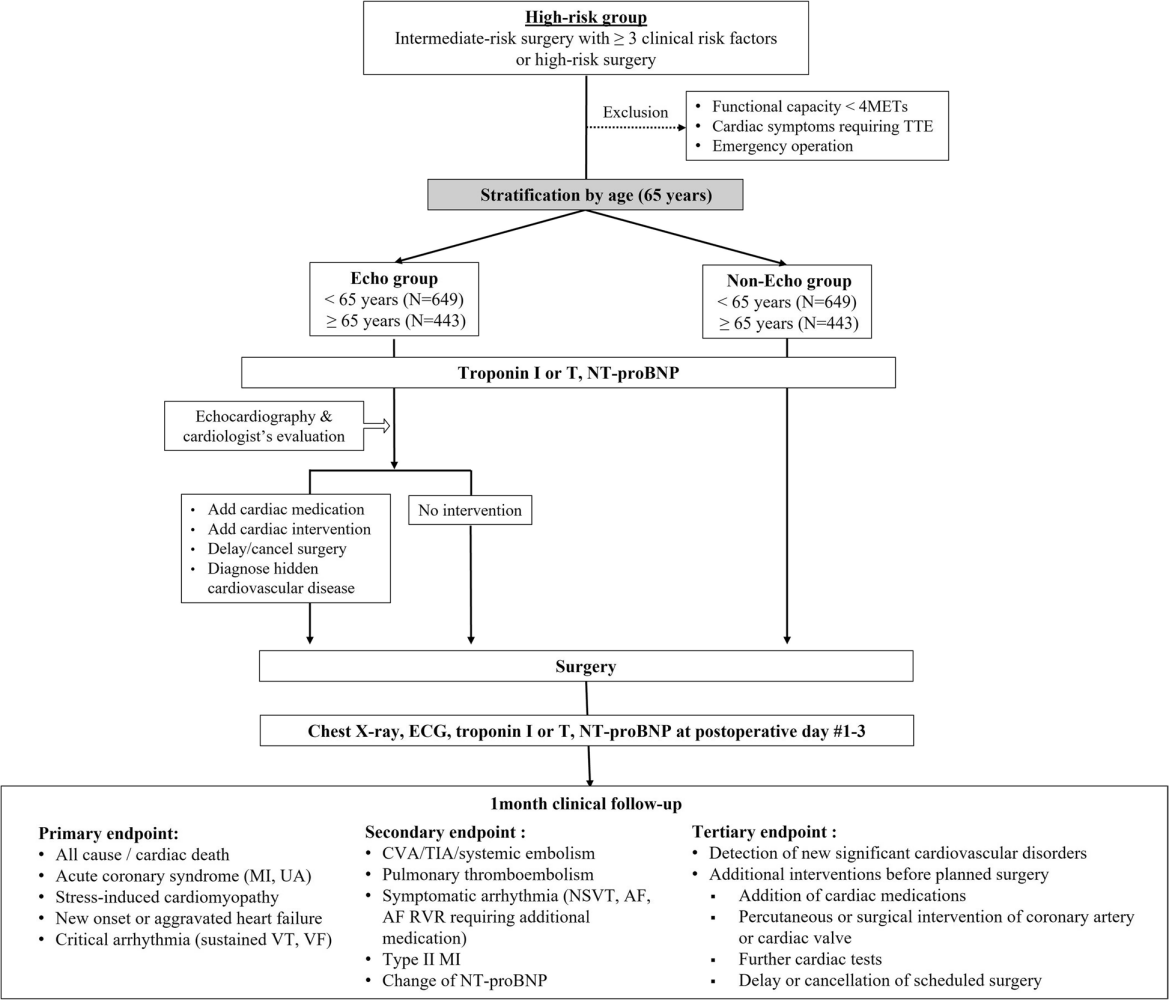
Study flow of the PREOP-ECHO trial: prospective cohort study for the high-risk group. Abbreviations. AF, atrial fibrillation; CVA, cerebrovascular accident; ECG, electrocardiography; MET, metabolic equivalent of task; MI, myocardial infarction; NSVT, nonsustained VT; NT-proBNP, N-terminal B-type natriuretic peptide; RVR, rapid ventricular response; TIA, transient ischemic attack; TTE, transthoracic echocardiography; VF, ventricular fibrillation; VT, ventricular tachycardia

**Fig. 3:**
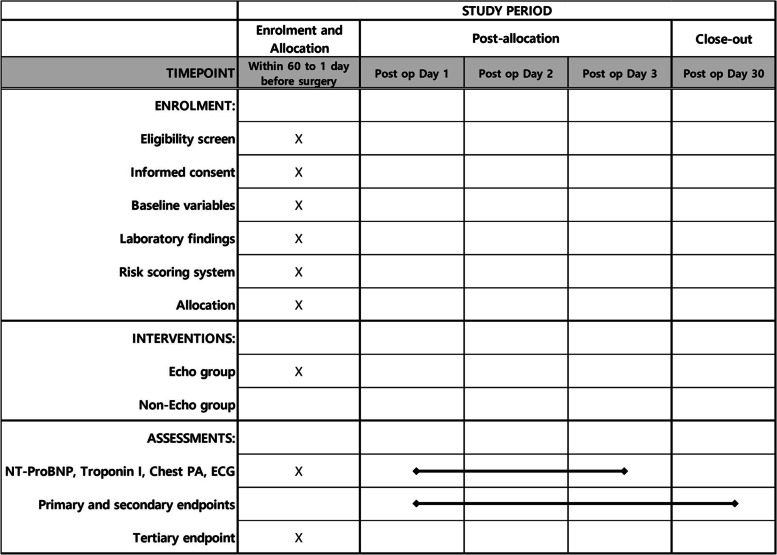
Schedule of enrollment, interventions, and assessments according to the Standard Protocol Items: Recommendations for Interventional Trials (SPIRIT) guideline. Abbreviations. ECG. Electrocardiography; NT-proBNP, N-terminal B-type natriuretic peptide

The first study is designed as a randomized controlled trial (RCT) for patients undergoing intermediate-risk surgery who have less than three clinical risk factors from the revised cardiac risk index (intermediate-risk group) (Table 1) [8, 11]. The second study is designed as a prospective cohort study for patients undergoing intermediate-risk surgery who have three or more clinical risk factors or for patients undergoing high-risk surgery regardless of the number of clinical risk factors (high-risk group) (Table 2). We re-classified surgical risk based on the guidelines from the European Society of Cardiology [8] and present specific surgeries in Table 3, including but not limited to the following representative examples. The study designs are shown in Figs. 1 and 2. This trial complied with Standard Protocol Items: Recommendations for Interventional Trials (SPIRIT) guidelines. The SPIRIT checklist can be found in Additional file 1, and the schedule for enrollment, interventions, and assessments is presented in Fig. 3.

**Table 1:** Clinical risk factors according to the revised cardiac risk index (Circulation, Lee 1999) [11]

• Ischemic heart disease (angina pectoris and/or previous myocardial infarction^a^) • Heart failure • Stroke or transient ischemic attack • Renal dysfunction (serum creatinine >170 umol/L or 2 mg/dL or a creatinine clearance of <60 mL/min/1.73m^2^) • Diabetes mellitus requiring insulin therapy	

^a^According to the universal definition of myocardial infarction

**Table 2:** Inclusion and exclusion criteria for the PREOP-ECHO trial

**Inclusion criteria**	
• Aged 18 to 90 years • Plan to undergo elective non-cardiac surgery	
**Randomized controlled trial** (Intermediate-risk group)	**Prospective cohort study** (high-risk group)
• Intermediate-risk surgery with less than three clinical risk factors	• Intermediate-risk surgery with three or more clinical risk factors or • High-risk surgery regardless of the number of clinical risk factors
**Exclusion criteria**	
• Current symptoms or signs requiring transthoracic echocardiography • Poor functional capacity (< 4 METs) • Life expectancy less than 6 months • Emergent surgery • Low-risk surgery • Participants who underwent TTE within 3 months before study enrollment	

*TTE* transthoracic echocardiography, *MET* metabolic equivalent of task

**Table 3:** Detailed examples of intermediate- and high-risk surgery

Intermediate-risk surgery	High-risk surgery
• Hip surgery	• Amputation (except toe/finger)
• Spine surgery (except vertebroplasty)	• Bowel perforation surgery
• Proximal bone surgery (including knee)	• Liver surgery
• Multiple bone fracture surgery	• Bile duct surgery
• Facial bone fracture surgery	• Duodeno-pancreas surgery
• Hollow viscus surgery (stomach, jejunum, colon, rectum)	• Arterial bypass surgery
• Hiatal hernia surgery	• Thoracic endovascular aortic repair
• Distal pancreas surgery	• Open thromboembolectomy
• Splenectomy	• Aorta, major vascular surgery
• Cholecystectomy	• Pneumonectomy
• Hysterectomy	• Lung transplantation
• Exploratory laparotomy	• Esophagectomy
• Nephrectomy	• Total cystectomy
• Kidney transplantation	• Adrenal resection
• Endovascular aortic repair	
• Lung resection	
• Neurosurgery (except transfemoral cerebral angiography)	
• Symptomatic carotid artery surgery	
• Neck surgery (except thyroid/parathyroid)

This multicenter prospective study is an investigator-initiated trial commenced by the Korean Society of Echocardiography. This study is supported by the Korea Health Industry Development Institute (KHIDI) and funded by the Ministry of Health & Welfare, Republic of Korea. The study will be conducted in 22 university hospitals in South Korea from May 2021, with a goal to complete enrollment in 2023.

### Study population

Patients aged 18 to 90 years who are scheduled to undergo elective intermediate- to high-risk noncardiac surgery are eligible for enrollment. According to predefined criteria, participants will be classified into the RCT for the intermediate-risk group or the prospective cohort study for the high-risk group. Patients with current symptoms or signs requiring TTE, patients with a poor functional capacity of less than four metabolic equivalent of task (METs), or patients with a life expectancy of less than 6 months will be excluded. Table 2 provides details on the inclusion and exclusion criteria.

### Randomization and stratification

Informed consent was achieved before screening and enrollment for all participants. Patients in the intermediate-risk group will be randomized to receive echocardiography or not, according to block randomization. An independent organization will generate a random table and assign randomization codes to the subjects using an interactive web response system of the Internet-based Clinical Research and Trial management system (iCReaT, version 2.0). The randomization will be performed on an institutional basis to prevent bias.

Patients in the high-risk group will be enrolled consecutively in the prospective cohort study irrespective of the implementation of preoperative echocardiography or not. The use of TTE is at the discretion of attending physicians. To reduce the risk of selection bias according to age, we will stratify the subjects according to the age of 65 years (Fig. 2).

For both studies, usual postoperative care including drug prescriptions or laboratory examinations can be performed as circumstances demand. Trial discontinuation or modifying allocated intervention can be permitted in case of the participants’ request or the medical need for echocardiography before surgery.

### Sample size calculation

The European Society of Cardiology guidelines indicate that the risk of MI and cardiac death within 30 days after surgery for the intermediate- and high-risk surgery cohorts are 1–5% and >5%, respectively [8]. As we defined the outcomes and the risk groups differently than in previous studies, we hypothesize that the incidence of the primary endpoint without preoperative echocardiography would be 4% in the intermediate-risk group. In the high-risk group, the risk of the primary endpoint was estimated to be 7% in patients under the age of 65 years old and 10% in patients older than 65 years old. The risk of the primary endpoint is assumed to be decreased by 50% in the preoperative echocardiography group. We calculated the requisite number of participants to reach a power of 80%, type I error of 5%, and a loss to follow-up of 2%. A total of 2,330 patients in the intermediate-risk group will be enrolled in the RCT (Fig. 1). For the prospective cohort study, 1,298 participants receiving preoperative TTE and 886 participants not receiving TTE are needed in each group stratified by age of 65 years (Fig. 2).

### Outcome definitions and follow-up

The primary endpoint of this study is a composite of the incidences of all-cause death, aborted sudden cardiac arrest, type I MI, unstable angina, stress-induced cardiomyopathy, lethal arrhythmias (such as sustained ventricular tachycardia or ventricular fibrillation), and newly diagnosed or acutely decompensated heart failure (HF). Type I MI is defined as an increase of cardiac troponin I or T above the 99th percentile of the upper reference limit and at least one evidence of coronary ischemia including symptoms, new ST-T change or pathologic Q wave on an electrocardiogram, new regional wall motion abnormality (RWMA), or coronary thrombus on angiography or autopsy according to the fourth universal definition of MI [19]. Because there is no unified definition of stress-induced cardiomyopathy, we modified the Mayo Clinic Diagnostic Criteria to include more strict standards [20, 21]: (1) new ST-segment or T wave change, (2) an increase in cardiac troponin greater than three times the upper reference limit, (3) new RWMA in TTE, and (4) no evidence of significant coronary artery disease explanatory of new RWMA by coronary angiography/coronary computed tomography or evidence of recovery of RWMA in the following TTE. Decompensated HF is diagnosed based on symptoms or signs presenting with pulmonary edema, cardiogenic shock, or abnormal systolic/diastolic dysfunction after surgery [22].

Secondary endpoints include documented incidences of symptomatic arrhythmias, cerebrovascular accidents, transient ischemic attack, systemic embolism, pulmonary embolism, type II MI, or elevated N-terminal pro B-type natriuretic peptide (NT-proBNP) above 300 pg/ml. Documented symptomatic arrhythmias are defined as nonsustained ventricular tachycardia, newly detected atrial fibrillation (AF), or rapid ventricular response requiring additional medical therapy in patients with well-controlled AF. Myocardial injury equivalent to type II MI is defined as elevated troponin levels greater than the 3-fold upper limit of the normal value [19].

The tertiary endpoint is preoperative detection of new significant cardiovascular disorders and/or performing additional intervention before the planned surgery that would not be conducted without the information from the preoperative TTE. Examples of newly diagnosed cardiovascular disorders include moderate or severe valvular heart disease, congenital heart disease, new RWMA suggesting ischemic heart disease, and cardiomyopathies, such as dilated cardiomyopathy, hypertrophic cardiomyopathy, noncompaction cardiomyopathy, sarcoidosis, or cardiac amyloidosis. Additional interventions include the addition of cardiac medications (e.g., nitrate, beta-blocker, vasodilator, or diuretics), percutaneous or surgical intervention of coronary artery or cardiac valves, further cardiac tests (e.g., exercise or pharmacologic stress test, coronary computed tomography, or coronary angiography), and delay/cancelation of scheduled surgery. If the participants are suspected to have significant cardiovascular disease based on the preoperative echocardiography, the next diagnostic step will be determined by physicians in the same manner as those used in routine clinical practice. Participants will be followed for events for 30 days after surgery by calls or visits.

### Data procurement

Demographic findings, comorbidities, and functional capacity will be prospectively collected before surgery. Electrocardiography, chest X-ray, and laboratory results, including troponin I or T and NT-proBNP levels, will be gathered preoperatively within 90 days before surgery and postoperatively within 3 days after surgery. The samples will be analyzed in the respective institutions. Information on surgery, such as operation time, amount of blood loss and blood transfusion, and anesthetic method, will be collected from operation records after surgery. All data will be anonymized and collected on structured case reporting forms (Additional file 2) and recorded in the iCReaT system provided by the Korea Disease Control and Prevention Agency.

To evaluate the discriminatory predictive power of preoperative echocardiography in the prediction of postoperative CVE compared with clinical risk factors, we will obtain traditional risk indices including Revised Cardiac Risk Index (RCRI) [11], Myocardial Infarction and Cardiac Arrest (MICA) score [10], and American College of Surgeons National Surgical Quality Improvement Program (ACS NSQIP) surgical risk calculator [23].

### Echocardiography

Transthoracic echocardiographic images and measurements will be obtained at individual hospitals according to guidelines from the American Society of Echocardiography [24]. Left ventricular dimensions are measured in the parasternal long-axis window. LV volumes and ejection fraction are estimated by modified Simpson’s method, and left atrial volume index is measured by the biplane area-length method and adjusted to body surface area. The presence of RWMA is evaluated based on the 17-segment model of the American Heart Association [25]. Indices of diastolic function will be obtained including the peak early diastolic trans-mitral flow velocity (E), the peak late diastolic trans-mitral flow velocity (A), E/A ratio, deceleration time, the early diastolic mitral annular velocity from the septal annulus (e′), E/e′ ratio, maximal velocity of tricuspid regurgitation, and size of inferior vena cava. To detect significant valvular heart disease, conventional or color Doppler interrogation to four cardiac valves will be performed. To measure LV global longitudinal strain, apical 4-, 2-, and 3-chamber images are acquired following the Digital Imaging and Communications in Medicine format and will be analyzed in the core laboratory by a specialist. All echocardiographic parameters obtained will be used in the post hoc analysis for the discovery of the potential predictor of postoperative CVE.

### Statistical analysis

#### RCT for intermediate-risk group

For continuous variables, data will be presented as means with standard deviation or medians with the interquartile range as appropriate. Categorical variables will be reported as the frequencies with percentiles. The baseline characteristics will be compared using the absolute standardized difference between the two groups. The variables will be perceived as imbalanced when an absolute standardized difference > 0.10. Univariable and multivariable logistic regression analysis will be used to identify significant independent variables associated with the development of endpoints. The effect of covariates on the outcome will be presented as odds ratio and 95% confidence intervals.

#### Prospective cohort study for the high-risk group

Baseline characteristics and the endpoints will be analyzed using the same analysis as RCT with correction for confounding variables. Because of the risk of selection bias, the primary endpoint will be analyzed additionally using propensity score matching as a sensitivity analysis.

All endpoints will be analyzed in the full analysis set and tested supplementally in the per-protocol set. The outcomes will be analyzed according to the assigned group, regardless of performing echocardiography or not, for the full analysis set (intention-to-treat analysis). For per-protocol analyses, subjects without endpoints or who violate the study protocol, such as inability to interpret echocardiography due to poor echo window, will be excluded. All individual components composing primary and secondary endpoints occurring within 30 days after index surgery are documented with the date of occurrence. Additional analysis of each individual component will be performed separately using logistic regression. Sensitivity analysis using the Fine-Gray model for the competing risk will be assessed for the competing risk of all-cause death [26].

Statistical analysis will be conducted using SAS (version 9.4, SAS Institute Inc., Cary, NC, USA). Findings with two-sided *p*-values less than 5% will be considered significant. All statistical analyses will be performed by the Biostatistics Collaboration Unit of Yonsei University College of Medicine.

### Oversight and monitoring

We have composed a study coordinating center, steering committee, endpoint adjudication committee, and data management team. The steering committee consists of E-YC, EKK, H-MC, and HSL. The study coordinating center is composed of a project manager and clinical research associates of a contract research organization (CRO), and the endpoint adjudication committee will be composed of principal investigators of each institution. The data management team consists of HSL, GP, and data monitoring associates of CRO. Each committee decides on the study protocol and performance after internal discussion and has responsibility for each study process. A data monitoring committee, which is independent of competing interests, will be composed to monitor data safety.

## Discussion

Recent healthcare guidelines generally do not support the routine use of preoperative echocardiography in asymptomatic patients due to a lack of the evidence of an additional benefit of TTE before noncardiac surgery. Per the European Society of Cardiology guidelines released in 2014, routine TTE before elective surgery is recommended only in patients with unstable cardiac conditions, such as unstable angina, acute HF, significant cardiac arrhythmia, symptomatic valvular heart disease, and myocardial infarction within the past 30 days with residual ischemia (COR Ic). In asymptomatic patients, resting echocardiography can be considered before high-risk surgery (COR IIb, LOE C) and is not recommended before low- to intermediate-risk surgery (COR III, LOE C) [8]. The American College of Cardiology/American Heart Association guidelines also indicate that routine preoperative evaluation of LV function is not recommended in asymptomatic patients [7]. British guidelines from the National Institute for Health and Care Excellence oppose resting echocardiography except for patients with a heart murmur, any cardiac symptom, or under the suspicion of HF [13]. The American Society of Echocardiography has defined preoperative echocardiography in the absence of symptoms or signs of cardiovascular disease as inappropriate use [27]. However, the incidence of valvular heart disease is increasing, especially in the aged population [28]. In a meta-analysis, a pooled prevalence of severe aortic stenosis in patients > 75 years of age was 3.4% [29]. Because patients undergoing elective surgery are getting older, the risk of encountering hidden valvular heart disease during surgery is expected to be increased [30]. Tashiro et al. demonstrated that the prevalence of 30-day major cardiovascular events was 18.2% of patients undergoing noncardiac surgery with severe aortic stenosis, while 10.5% in patients without severe aortic stenosis [31]. Even in asymptomatic severe valvular heart disease, patients with pulmonary arterial pressure over 50 mmHg or LV systolic dysfunction are recommended to undergo valvular intervention or optimization of loading conditions under special hemodynamic monitoring during the perioperative period [30].

Therefore, it is time to reevaluate the usefulness and scope of preoperative echocardiography for various reasons. First, commonly used preoperative risk calculators do not incorporate echocardiographic parameters in their scoring system, despite the wide use of preoperative echocardiography in the real world [7, 8]. Second, postoperative outcomes are becoming more complicated. Although the risk of perioperative complications, especially those that can be fatal, is decreasing in part due to quality improvement and advancement of surgical and anesthetic manipulations [2, 3], patients undergoing noncardiac surgeries are getting older. As the age of the patient increases, it is natural to have more comorbidities related to cardiovascular disease. However, available risk assessment systems do not account for postoperative heart failure, systemic embolism, pulmonary embolism, cerebrovascular accident, and atrial fibrillation, even though the prevalences thereof are increasing in aging societies [32, 33]. Therefore, it is important to also consider the likelihood of new postoperative CVEs that can increase medical costs and cause further debilitation or frailty in these patients. Although the ACS NSQIP provides information on the risk of cardiac complications and venous thromboembolism, the other outcomes mainly focus on the complications of surgery, not on the CVEs [34]. Third, the use of preoperative echocardiography has been drastically increased without scientific evidence supporting the benefits in reducing postoperative CVE. Issues concerning medical costs and inappropriate distribution of healthcare resources might arise because the possibility to discover significant abnormality is low when there is no suspicion of structural heart abnormality [35]. Therefore, it is necessary to investigate the predictive value of echocardiographic parameters and integrate them into the traditional clinical risk stratification system.

In the present study, we provide new classifications of risk groups. Risk is redefined by merging the surgical risk and the clinical risk of individual participants. We also classified surgical risk more thoroughly by incorporating the organ system undergoing surgery and the known risk of individual surgery. With more detailed risk classifications, we expect more accurate postoperative risk assessment for individual surgeries. Moreover, less severe, but clinically significant, endpoints such as heart failure, stress-induced cardiomyopathy, cerebrovascular accidents, pulmonary embolism, and atrial fibrillation, are also included in this study; thus, we may be able to discover new common postoperative CVEs in the current medical landscape. To the best of our knowledge, there have been no large-scale RCTs or prospective cohort studies investigating the benefits of preoperative echocardiography. The results of this study may provide information on specific groups of patients who would benefit from preoperative echocardiography before noncardiac surgery. Additionally, all patients enrolled in the RCT and prospective cohort studies will undergo preoperative plasma NT-proBNP and troponin I or T evaluation. These biomarkers will be used for the assessment of primary and secondary outcomes as well as the possible predictor of the postoperative CVE risk.

In summary, the PREOP-ECHO trial will gather data regarding the use of preoperative echocardiography in patients undergoing intermediate- to high-risk noncardiac surgery. The results will help in developing models to predict the risk of postoperative CVE and expand postoperative CVEs to include heart failure, nonfatal arrhythmia, and stress-induced cardiomyopathy.

## Trial status

This trial is registered at the Clinical Research Information Service KCT0006279 for RCT and KCT0006280 for a prospective cohort study on June 21, 2021. The enrollment was started in May 2021. Recruitment is expected to be completed in June 2023. Current protocol version and date: V1.3, date May 18, 2021.

## Supplementary Information

**Additional file 1.** SPIRIT checklist for the PREOP-ECHO trial.

**Additional file 2.** Case reporting form of the PREOP-ECHO trial.

## Data Availability

The final dataset will be available after completion of the trial from the corresponding author upon reasonable request under permission of the National Evidence-based healthcare Collaborating Agency.

## Abbreviations

ACS NSQIP: American College of Surgeons National Surgical Quality Improvement Program
AF: Atrial fibrillation
COR: Class of recommendation
CVE: Cardiovascular events
HF: Heart failure
LOE: Level of evidence
LV: Left ventricular
MET: Metabolic equivalent of task
MI: Myocardial infarction
MICA: Myocardial Infarction and Cardiac Arrest
NT-proBNP: N-terminal pro B-type natriuretic peptide
TTE: Transthoracic echocardiography
RCT: Randomized controlled trial
RWMA: Regional wall motion abnormality
RCRI: Revised Cardiac Risk Index
SPIRIT: Standard Protocol Items Recommendations for Interventional Trials
