# Supplementary material for: PRE-OPerative ECHOcardiograhy for prevention of cardiovascular events after non-cardiac surgery in intermediate- and high-risk patients: protocol for a low-interventional, mixed-cohort prospective study design (PREOP-ECHO)
Source: Trials. 2022 Sep 14;23:776. doi: 10.1186/s13063-022-06701-2 (PMC9476301; doi:10.1186/s13063-022-06701-2)
Supplement: Supplementary file 2 — Additional file 2. Case reporting form of the PREOP-ECHO trial. [file 13063_2022_6701_MOESM2_ESM.doc]

**Case Report Form**

**Title of study: The role of PRE-OPerative ECHOcardiography for the prevention of cardiovascular events after noncardiac surgery (PREOP-ECHO)**

Participant information

| **Name of institution** |  |
| --- | --- |
| **Name of participant (Initial)** |  |
| **Birth date** |  |
| **Sex** | ○ Male ○ Female ○ Unknown |
| **Date of written consent** |  |

| **Screening visit: from 60 days to 1 day before index surgery** | | | |
| --- | --- | --- | --- |
| **Date of visit**  **(YYYY-MM-DD)** | **Code of Participant** | **Initial of participant** | **Signature of PI** |
|  |  |  |  |

**1**. Patient characteristics

| Height (cm) |  | Weight (kg) |  |
| --- | --- | --- | --- |
| Blood Pressure | / mmHg | Pulse rate | bpm |
| Clinical diagnosis (Purpose of surgery) |  | | |
| Information on surgery | 1) Name of surgery: _________________________________________________  Approach: □ Open □ Laparoscopic or VATS □ Robotic  2) Location of surgical procedure  □ Anorectal □ Aortic □ Bariatric □ Brain □ Breast  □ ENT (except thyroid/parathyroid)  □ Foregut/Hepatobiliary □ GB, appendix, adrenal, spleen  □ Hernia (ventral, inguinal, femoral) □ Intestinal  □ Neck (Thyroid/parathyroid) □ Obstetric/Gynecologic  □ Other abdominal □ Peripheral vascular □ Spine  □ Non-esophageal thoracic □ Vein □ Urology    3) Classification of American Society of Anesthesiologists (ASA)  □ I. Healthy  □ II. Mild systemic disease, no functional limitations  □ III. Moderate to severe systemic disease, functional limitations  □ IV. Severe systemic disease, constantly life threatening, functionally. incapacitating  □ V. Not expected to survive with or without surgery 24h  4) Clinical risk factors according to the revised cardiac risk index  □ Ischemic heart disease (angina pectoris and/or previous myocardial infarction*)  □ Heart failure  □ Stroke or transient ischemic attack  □ Renal dysfunction (serum creatinine >170 umol/L or 2 mg/dL or a creatinine clearance of <60 mL/min/1.73m2)  □ Diabetes mellitus requiring insulin therapy  *According to the universal definition of myocardial infarction | | |

**2. Comorbidities**

| ⬜ Hypertension | ⬜ Controlled ⬜ Uncontrolled | |
| --- | --- | --- |
| ⬜ Smoking | ⬜ Non-smoker⬜ Ex-smoker:⬜ Current smoker: | |
| ⬜ Alcohol | ⬜ Non-drinker ⬜ Ex-drinker ⬜ Current drinker | |
| ⬜ DM | ⬜ OHA ⬜ Insulin ⬜ Non-drug Tx. | |
| ⬜ Hypercholesterolemia or low HDL | | |
| ⬜ Menopause without HRT | | |
| ⬜ Family history of CAD | | |
| ⬜ Peripheral vascular disease | | |
| ⬜ Cerebrovascular disease | | |
| ⬜ Chronic kidney disease (Last Creatinine: ______ mg/dl) | | ⬜ Dialysis |
| ⬜ Congestive heart failure  If yes: ⬜ <1month ⬜ <3months ⬜ >3months | | |
| ⬜ Severe COPD ⬜ Sepsis (within 48 hours before surgery) ⬜ Mechanical ventilation  ⬜ Disseminated cancer ⬜ Ascites (within 30 days before surgery) ⬜ Chronic steroid user | | |
| Functional status:  4-10 METs  > 10 METs   | 1 MET  🡫 | Self-care  Eating, dressing, or using the toilet  Walking indoors and around the house  Walking one to two blocks on level ground at 2 to 3 mph | | --- | --- | | 4 METs  🡫 | Light housework (e.g., dusting, washing dishes)  Climbing a flight of stairs or walking up a hill  Walking on level ground at 4 mph  Running a short distance  Heavy housework (e.g., scrubbing floors, moving heavy furniture)  Moderate recreational activities (e.g., golf, dancing, doubles tennis, throwing a baseball or football) | | 10 METs  🡫 | Strenuous sports (e.g., swimming, singles tennis, football, basketball, skiing) | | | |

**3. Detailed information on comorbidities (within 1 year before screening): □ No □ Yes**

| No | Diagnoiss | State of disease^1)^ | Date of diagnosis  (YYYY-MM-DD) | | Treatment | Treatment within 1 year^2)^ | Note |
| --- | --- | --- | --- | --- | --- | --- | --- |
| 1 |  |  |  | |  | ⬜ Yes |  |
| 2 |  |  |  | |  | ⬜ Yes |  |
| 3 |  |  |  | |  | ⬜ Yes |  |
| 4 |  |  |  | |  | ⬜ Yes |  |
| 5 |  |  |  | |  | ⬜ Yes |  |
| ^1)^State of disease  1 Before  2 After  3 Coincident  4 Ongoing  5 Unknown | | | | ^2)^Treatment within 1 year  0 No  1 Yes  2 Unknown | | | |

**4. Medication (Within 3 months before screening visit): □ No □ Yes**

| No | **Generic name** | **Daily dose** | **Unit (mg)** | **Taking currently** | **Note** |
| --- | --- | --- | --- | --- | --- |
| 1 |  |  |  | ⬜ Yes |  |
| 2 |  |  |  | ⬜ Yes |  |
| 3 |  |  |  | ⬜ Yes |  |
| 4 |  |  |  | ⬜ Yes |  |
| 5 |  |  |  | ⬜ Yes |  |

**5. Family history of cardiovascular diseases: □ No □ Yes**

| No | Relation | Diagnosis | Treatment | Note |
| --- | --- | --- | --- | --- |
| 1 |  |  |  |  |
| 2 |  |  |  |  |
| 3 |  |  |  |  |
| 4 |  |  |  |  |
| 5 |  |  |  |  |

**6. Electrocardiography** ⬜ **Not done** ⬜ **Done**

**Date of examination (YYYY-MM-DD): - -**

| ⬜ Normal sinus rhythm | |
| --- | --- |
| ⬜ Atrial fibrillation/flutter ⬜ Ventricular premature beat ⬜ pacemaker rhythm | |
| ⬜ Others | Comment: |

**7. Chest X-ray** ⬜ **Not done** ⬜ **Done**

**Date of examination (YYYY-MM-DD): - -**

| ⬜ Normal | |
| --- | --- |
| ⬜ Abnormal | Comment |

**8. Laboratory test** ⬜ **Not done** ⬜ **Done**

**Date of examination (YYYY-MM-DD): - -**

| **Category** | **Unit** | **Results** | **Category** | **Unit** | **Results** |
| --- | --- | --- | --- | --- | --- |
| ⬜ cTnT or ⬜ TnI | ng/ml* |  | NT-proBNP | pg/ml |  |
| AST | IU/L |  | Na | mmol/L |  |
| ALT | IU/L |  | K | mmol/L |  |
| Glucose (fasting) | mg/dL |  | BUN | mg/dL |  |
|  |  |  | Creatinine | mg/dL |  |

*Caution needed for different units according to the individual institution

**9. Echocardiography** ⬜ **Not done** ⬜ **Done**

**Date of examination (YYYY-MM-DD): - -**

| **Category** | **Unit** | **Results** | **Category** | **Unit** | **Results** |
| --- | --- | --- | --- | --- | --- |
| LV end diastolic dimension | mm |  | E | m/sec |  |
| LV end systolic dimension | mm |  | A | m/sec |  |
| LV ejection fraction by Modified Simpson’s method | % |  | E/A | - |  |
| Interventricular septal thickness (end-diastolic) | mm |  | Septal e’ | m/sec |  |
| LV posterior wall thickness (end-diastolic) | mm |  | Septal a’ | m/sec |  |
| Deceleration time | msec |  | E/e’(septal) | - |  |
| LA volume index  ⬜ Biplane area-length method (preferred)  ⬜ Biplane Simpson’s method  ⬜ Ellipsoid method | ml/m^2^ |  | TR Vmax | m/sec |  |
| Regional wall motion abnormality | Y/N |  | IVC size | mm |  |
| Global longitudinal strain  ⬜ GE  ⬜ Philips/Tomtec  ⬜ Siemens | % |  | RA pressure | mmHg |  |
| Significant valve disease  (moderate or severe) | ⬜ No ⬜ Yes | If Yes, please explain.  ⬜ AS ⬜ MS ⬜ MR ⬜ AR ⬜ TR | | | |

**10. Risk Calculator**

| Revised Cardiac Risk Index ([RCRI](https://qxmd.com/calculate/calculator_195/revised-cardiac-risk-index-lee-criteria)) | □ ___ points (0-6)  □ 30-day risk of major cardiac event ____ % |
| --- | --- |
| Gupta Risk for Myocardial Infarction or Cardiac Arrest ([MICA](https://qxmd.com/calculate/calculator_245/gupta-perioperative-cardiac-risk)) | □ ______ % |
| ACS [NSQIP](https://riskcalculator.facs.org/RiskCalculator/)  - Serious complication  - Cardiac complication  - Length of hospital day | □ _____ %  □ _____ %  □ _____ days |

**11.** Differential Diagnosis

| Stress echocardiography (exercise or pharmacologic) | ⬜ Done ⬜ not Done |
| --- | --- |
| Coronary computed tomography | ⬜ Done ⬜ not Done |
| Coronary angiography | ⬜ Done ⬜ not Done |
| **Significant coronary disease^1)^** | ⬜ Yes ⬜ No |
| **Special consultation to cardiologist^2)^** | ⬜ Yes ⬜ No |
| Comment |  |

^1)^ Criteria for significant coronary disease

- Stress echocardiography: New regional wall motion abnormality under exercise or pharmacologic stress

- Coronary computed tomography or coronary angiography: More than 70% stenosis of at least one epicardial coronary artery (Except for the patent stent of previous coronary stent)

^2)^ Special consultation to cardiologists: Need additional unplanned consultation to cardiologists due to the results of preoperative examination, irrespective of the usage of echocardiography

**12. Exercise Test** ⬜ **Not done** ⬜ **Done**

**Date of examination (YYYY-MM-DD): - -**

| Cardiopulmonary exercise test | VO_2max_ _________mL/Kg/min  VO_2max_/Pre% _________%  VO_2max_ METS _________METS |
| --- | --- |

**13. Inclusion and exclusion criteria**

| **Substudy 1: Prospective randomized-controlled trial for intermediate-risk group** | | | |
| --- | --- | --- | --- |
| **Inclusion criteria** | |  | |
| 1 | Age from 18 to 90 | ⬜ No ⬜ Yes | |
| 2 | Plan to undergo elective intermediate-risk non-cardiac surgery by ESC/AHA guideline and having less than three clinical risk factors | ⬜ No ⬜ Yes | |
| 3 | No current symptoms or signs requiring transthoracic echocardiography | ⬜ No ⬜ Yes | |
| **Exclusion criteria** | |  |  |
| 1 | Plan to undergo elective low- or high-risk non-cardiac surgery by ESC/AHA guideline | ⬜ No ⬜ Yes | |
| 2 | Poor functional capacity (< 4METs) | ⬜ No ⬜ Yes | |
| 3 | Life expectancy less than 6 months | ⬜ No ⬜ Yes | |
| 4 | Emergent surgery | ⬜ No ⬜ Yes | |
| 5 | Having results of transthoracic echocardiography within 3 months before study enrollment | ⬜ No ⬜ Yes | |
| **Is this participant suitable for this study?** | | **⬜ No ⬜ Yes** | |

| **Substudy 2: Prospective cohort study for high-risk group** | |  | |
| --- | --- | --- | --- |
| **Inclusion criteria** | |  | |
| 1 | Age from 18 to 90 | ⬜ No ⬜ Yes | |
| 2 | Plan to undergo elective non-cardiac surgery   - Intermediate-risk surgery by ESC/AHA guideline and having three or more clinical risk factors, or - High-risk surgery regardless of the number of clinical risk factors | ⬜ No ⬜ Yes | |
| 3 | No current symptoms or signs requiring transthoracic echocardiography | ⬜ No ⬜ Yes | |
| **Exclusion criteria** | |  |  |
| 1 | Plan to undergo elective low-risk non-cardiac surgery by ESC/AHA guideline | ⬜ No ⬜ Yes | |
| 2 | Poor functional capacity (< 4METs) | ⬜ No ⬜ Yes | |
| 3 | Life expectancy less than 6 months | ⬜ No ⬜ Yes | |
| 4 | Emergent surgery | ⬜ No ⬜ Yes | |
| **Is this participant suitable for this study?** | | **⬜ No ⬜ Yes** | |

**14. Event*** ⬜ No ⬜ Yes If Yes,

*Events occurred from acquisition of written consent before index surgery

| ⬜ Death  **Cause of death**: ⬜ cardiac ⬜ Non-cardiac  If Non-cardiac, explain: | Date (YYYY/MM/DD): / / |
| --- | --- |
| ⬜ Acute MI | Date (YYYY/MM/DD): / / |
| ⬜ Unstable angina | Date (YYYY/MM/DD): / / |
| ⬜ Stress induced cardiomyopathy | Date (YYYY/MM/DD): / / |
| ⬜ Clinical HF (e.g. pulmonary edema) | Date (YYYY/MM/DD): / / |
| ⬜ Symptomatic documented arrhythmia  ⬜ Sustained VT  ⬜ VF  ⬜ Non-sustained VT  ⬜ New onset AF (in whom having sinus rhythm before surgery)  ⬜ AF RVR requiring medication (in whom having AF before surgery) | Date (YYYY/MM/DD): / /  Date (YYYY/MM/DD): / /  Date (YYYY/MM/DD): / /  Date (YYYY/MM/DD): / /  Date (YYYY/MM/DD): / / |
| ⬜ CVA/TIA/systemic embolism | Date (YYYY/MM/DD): / / |
| ⬜ PTE | Date (YYYY/MM/DD): / / |

*MI, myocardial infarction; UA, unstable anginal; VT, ventricular tachycardia; VF, ventricular fibrillation; CVA, cerebrovascular event; TIA, transient ischemic attack; NSVT, nonsustained ventricular tachycardia; AF, atrial fibrillation; RVR, rapid ventricular response

**15**. Cardiologist’s evaluation and management

| ⬜ Add cardioprotective medications  ⬜ Beta-blocker ⬜ Nitrate ⬜ Diuretics ⬜ Others  Comment: | Date (YYYY/MM/DD): / / |
| --- | --- |
| ⬜ Further cardiac function evaluation  Comment: | Date (YYYY/MM/DD): / / |
| ⬜ Delay/Cancel surgery  Comment: | Date (YYYY/MM/DD): / / |
| ⬜ Cardiac Intervention  Comment: | Date (YYYY/MM/DD): / / |
| ⬜ Detection of new cardiovascular disease  ⬜ Moderate or severe degree valvular heart disease  ⬜ Congenital heart disease (atrial septal defect, ventricular septal defect, patent ductus arteriosus, Ebstein’s anomaly)  ⬜ Cardiomyopathy (hypertrophic cardiomyopathy, dilated cardiomyopathy, noncompaction cardiomyopathy, sarcoidosis, cardiac amyloidosis)  ⬜ Newly detected regional wall motion abnormality from ischemic heart disease  ⬜ Others  Comment: | Date (YYYY/MM/DD): / /  Date (YYYY/MM/DD): / /  Date (YYYY/MM/DD): / /  Date (YYYY/MM/DD): / /  Date (YYYY/MM/DD): / / |

| **After surgery: from 1 to 3 days after index surgery** | | | |
| --- | --- | --- | --- |
| **Date of visit**  **(YYYY-MM-DD)** | **Code of Participant** | **Initial of participant** | **Signature of PI** |
|  |  |  |  |

1. Patient characteristics

| **Blood Pressure** | / mmHg | **Pulse rate** | bpm | **Weight** | kg |
| --- | --- | --- | --- | --- | --- |

**2. Electrocardiography** ⬜ **Not done** ⬜ **Done**

**Date of examination (YYYY-MM-DD): - -**

| ⬜ Normal sinus rhythm | |
| --- | --- |
| ⬜ Atrial fibrillation/flutter ⬜ Ventricular premature beat ⬜ pacemaker rhythm | |
| ⬜ Others | Comment: |

**3. Chest X-ray** ⬜ **Not done** ⬜ **Done**

**Date of examination (YYYY-MM-DD): - -**

| ⬜ Normal | |
| --- | --- |
| ⬜ Abnormal | Comment |

**4. Laboratory test** ⬜ **Not done** ⬜ **Done**

**Date of examination (YYYY-MM-DD): - -**

| **Category** | **Unit** | **Results** | **Category** | **Unit** | **Results** |
| --- | --- | --- | --- | --- | --- |
| ⬜ cTnT or ⬜ TnI | ng/ml* |  | NT-proBNP | pg/ml |  |
| AST | IU/L |  | Na | mmol/L |  |
| ALT | IU/L |  | K | mmol/L |  |
| Glucose (fasting) | mg/dL |  | BUN | mg/dL |  |
|  |  |  | Creatinine | mg/dL |  |

*Caution needed for different units according to the individual institution

**5. Information on surgery**

| Date of surgery (YYYY/MM/DD) | /  **/** |
| --- | --- |
| Amount of RBC* transfusion during surgery (pint) |  |
| Estimated blood loss during sugery (ml) |  |
| Anesthesia time (minute) |  |
| Anesthesia method | ⬜ General anesthesia ( ⬜ Inhalation ⬜ Intravenous)  ⬜ Spinal anesthesia  ⬜ MAC* or others (Comment: ) |
| Surgery time (minute) |  |
| Name of surgery (from surgical record) |  |
| Final diagnosis (postoperative) |  |

*RBC, red blood cell; MAC, monitored anesthesia care

**6. Event**  No  Yes If Yes,

| ⬜ Death  **Cause of death**: ⬜ cardiac ⬜ Non-cardiac  If Non-cardiac, explain: | Date (YYYY/MM/DD): / / |
| --- | --- |
| ⬜ Acute MI | Date (YYYY/MM/DD): / / |
| ⬜ Unstable angina | Date (YYYY/MM/DD): / / |
| ⬜ Stress induced cardiomyopathy | Date (YYYY/MM/DD): / / |
| ⬜ Clinical HF (e.g. pulmonary edema) | Date (YYYY/MM/DD): / / |
| ⬜ Symptomatic documented arrhythmia  ⬜ Sustained VT  ⬜ VF  ⬜ Non-sustained VT  ⬜ New onset AF (in whom having sinus rhythm before surgery)  ⬜ AF RVR requiring medication (in whom having AF before surgery) | Date (YYYY/MM/DD): / /  Date (YYYY/MM/DD): / /  Date (YYYY/MM/DD): / /  Date (YYYY/MM/DD): / /  Date (YYYY/MM/DD): / / |
| ⬜ CVA/TIA/systemic embolism | Date (YYYY/MM/DD): / / |
| ⬜ PTE | Date (YYYY/MM/DD): / / |

*MI, myocardial infarction; UA, unstable anginal; VT, ventricular tachycardia; VF, ventricular fibrillation; CVA, cerebrovascular event; TIA, transient ischemic attack; NSVT, nonsustained ventricular tachycardia; AF, atrial fibrillation; RVR, rapid ventricular response

| **After surgery: Within 30 ± 10 days after index surgery** | | | |
| --- | --- | --- | --- |
| **Date of visit**  **(YYYY-MM-DD)** | **Code of Participant** | **Initial of participant** | **Signature of PI** |
|  |  |  |  |

**1. Event**  No  Yes If Yes,

| ⬜ Death  Cause of death: ⬜ cardiac ⬜ Non-cardiac  If Non-cardiac, explain: | Date (YYYY/MM/DD): / / |
| --- | --- |
| ⬜ Acute MI | Date (YYYY/MM/DD): / / |
| ⬜ Unstable angina | Date (YYYY/MM/DD): / / |
| ⬜ Stress induced cardiomyopathy | Date (YYYY/MM/DD): / / |
| ⬜ Clinical HF (e.g. pulmonary edema) | Date (YYYY/MM/DD): / / |
| ⬜ Symptomatic documented arrhythmia  ⬜ Sustained VT  ⬜ VF  ⬜ Non-sustained VT  ⬜ New onset AF (in whom having sinus rhythm before surgery)  ⬜ AF RVR requiring medication (in whom having AF before surgery) | Date (YYYY/MM/DD): / /  Date (YYYY/MM/DD): / /  Date (YYYY/MM/DD): / /  Date (YYYY/MM/DD): / /  Date (YYYY/MM/DD): / / |
| ⬜ CVA/TIA/systemic embolism | Date (YYYY/MM/DD): / / |
| ⬜ PTE | Date (YYYY/MM/DD): / / |

*MI, myocardial infarction; UA, unstable anginal; VT, ventricular tachycardia; VF, ventricular fibrillation; CVA, cerebrovascular event; TIA, transient ischemic attack; NSVT, nonsustained ventricular tachycardia; AF, atrial fibrillation; RVR, rapid ventricular response

| **End of study** | | | |
| --- | --- | --- | --- |
| **Date of visit**  **(YYYY-MM-DD)** | **Code of Participant** | **Initial of participant** | **Signature of PI** |
|  |  |  |  |

| **Completion date of study (YYYY/MM/DD)** | /  **/** |
| --- | --- |
| **Is the participant complete the study according to the study protocol?** | ⬜ Yes ⬜ No |
| **If “No”, Date of dropout (YYYY/MM/DD)** | /  **/** |
| **Cause of dropout or early termination** | |
| ⬜ Withdraw consent  ⬜ Violate inclusion/exclusion criteria  ⬜ Adverse event  ⬜ Do not visit or answer the phone after surgery  ⬜ Violate study protocol  ⬜ Cannot proceed the study assessed by investigator  ⬜ Others (Comment: ) | |
